# The future of inguinal Lymphadenecotmy in penile cancer: laparoscopic or robotic?

**DOI:** 10.1590/S1677-5538.IBJU.2019.02.01

**Published:** 2019-04-01

**Affiliations:** 1 Unidade de Pesquisa Urogenital da Univ. Estadual do Rio de Janeiro – UERJ, Rio de Janeiro, RJ, Brasil;; 2 Hospital Federal da Lagoa, Rio de Janeiro, RJ, Brasil;; 3 Editor Associado da International Braz J Urol

The March-April 2019 issue of the International Braz J Urol presents original contributions with a lot of interesting papers in different fields: Prostate Cancer, Renal stones, Renal Cell Carcinoma, Bladder Cancer, Uretrhal Strictures, Trauma, Prostate Biopsy, Kidney Transplant, neurogenic Bladder and Penile Cancer. The papers come from many different countries such as Brazil, USA, Turkey, China, Italy, Iran, Argentina, Spain, South Korea, and United Kingdon, and as usual the editor's comment highlights some papers. We decided to comment the paper about a very interesting topic: The treatment of the inguinal lymph nodes in penile cancer.

Doctor Meneses and collegues from Brazil performed on page 325 an interesting study about the Video Endoscopic management of inguinal lympadenectomy in penile cancer. The authors described the initial experience with this method and analyzed the post-surgical complications in 11 patients with penile cancer (stages T2 or T3). They observed the bleeding, drainage time, cellulitis, lymphocele, cutaneous necrosis, miocutaneous necrosis and hospitalization time. The results of the paper shows that no patient showed intrasurgical complications, bleeding > 50 mL or conversion. The global complication rate was 33.2% (27.2% were lymphatic). No patient showed cutaneous necrosis. The authors concluded that video endoscopic management of inguinal lympadenectomy in penile cancer is a safe and easy technique with lower incidence of complications.

Malignant neoplasm of the penis is a rare disease, being more common in regions with low socioeconomic levels, accounting for approximately 2% of malignancies in man, with squamous cell carcinoma (SCC) being the most common type (1, 2). Considering that tumor dissemination is preferentially done lymphatic (initially for superficial inguinal lymph nodes and later for deep inguinal and pelvic lymph nodes), the presence of metastases in the inguinal lymph nodes is the main variable capable of affecting the survival in these patients (3). In this way, bilateral inguinal lymphadenectomy represents the only procedure capable of identifying and treating inguinal micrometastases early, although its prophylactic indication is controversial in the literature (4–6). The following are the main indications of lymphadenectomy: tumors > 2 cm, high-grade tumors (histopathological grade II or III), advanced local staging (T2-T4), lymphovascular microscopic invasion, palpable inguinal lymph nodes after antibiotic therapy, palpable inguinal lymph nodes that appeared in the follow-up without evidence of distant disease and unsatisfactory clinical evaluation (obese, inguinal surgery) (4).

Inguinal lymphadenectomy represents an important stage of treatment. However, it should be noted that about 50% of patients submitted to open inguinal lymphadenectomy have important complications, such as wound infection (26%), necrosis and dehiscence of operative wound (41%) and lymphocele (21%) thus being a procedure with high morbidity (5, 6). The paper of Meneses and collegues shows that laparoscopic video technique is a very good option, but the authors had 30% of complications. In a recent paper where the outcomes between open and robotic surgery were compared a multivariable analysis shows that the pathological nodal stage and open inguinal lymph node dissection were the independent risk factors associated with an increased risk of major complications (7). A systematic review published in the present year shows lower rates of complications of robotic surgery compared with open surgery (8).

We need more evidences, but we can conclude that robotic surgery will be the gold standard treatment for inguinal lymphadenectomy in penile cancer.
